# Relationship Between Short-Term Blood Pressure Variability and Choroidal–Retinal Thicknesses Assessed by Optical Coherence Tomography in Hypertensive Subjects

**DOI:** 10.3390/jpm14121123

**Published:** 2024-11-27

**Authors:** Caterina Carollo, Maria Vadalà, Marta Ferrara, Enea Chisci, Alberto La Felice, Katia Valeria Di Natale, Alessandra Sorce, Vincenza Maria Elena Bonfiglio, Giuseppe Mulè

**Affiliations:** 1 Unit of Nephrology and Dialysis, Hypertension Excellence Centre, Department of Health Promotion, Mother and Child Care, Internal Medicine and Medical Specialties (PROMISE), Università di Palermo, 90128 Palermo, Italy; marta.ferrara@unito.it (M.F.); avinouros85@gmail.com (A.S.); giuseppe.mule@unipa.it (G.M.); 2 Biomedicine, Neuroscience and Advance Diagnostic (BIND) Department, University of Palermo, 90128 Palermo, Italy; maria.vadala@unipa.it (M.V.); enea.chisci@unipa.it (E.C.); alberto.lafelice@unipa.it (A.L.F.); vincenzamariaelena.bonfiglio@unipa.it (V.M.E.B.); 3 Unit of Pediatric Nephrology, ARNAS CIVICO, 90127 Palermo, Italy; katiav.dinatale@gmail.com

**Keywords:** choroidal thickness, blood pressure variability, hypertension, microcirculation

## Abstract

**Background/Objectives:** The complications of hypertension depend not only on the mean blood pressure (BP) but also on its variability (BPV). Recent studies suggest that the choroid may serve as an indicator of systemic vascular damage. These studies have been made possible by the increased availability of optical coherence tomography (OCT). The aim of our study was to analyze the relationship between short-term BP variability (STBPV) and choroid–retinal thickness in hypertensive patients. **Methods:** A total of 98 patients with a mean age of 49 ± 12 years were enrolled in the study. All participants underwent 24 h blood pressure (BP) monitoring to measure 24 h mean systolic (SBP) and diastolic blood pressure (DBP), along with their respective standard deviations (SD), the weighted SD of 24 h SBP and DBP, and the average real variability (ARV) of 24 h SBP and DBP. The choroid–retinal region was assessed using Swept-Source OCT, with choroidal thickness (ChT) and retinal thickness divided into three concentric rings, and their mean choroidal thickness (ChT-or) was calculated. **Results:** The choroidal thickness of the concentric rings was found to be inversely correlated with all ARV values of the monitored blood pressure means. In particular, a correlation was observed between the ARV of daytime DBP and ChT-or. This correlation remained statistically significant (β = −0.34; *p* = 0.02) even after adjustment for various confounding factors. The ARV of daytime DBP was the only STBPV index to maintain a significant association, in the multivariate analysis, with the central ring mean thickness (β = −0.314; *p* = 0.001) and the inner choroidal ring mean thickness (β = −0.262; *p* = 0.003). **Conclusions:** Our study demonstrated an independent negative association between short-term BP variability (STBPV), when expressed as ARV of daytime DBP, and choroidal thickness. This finding confirms the value of choroidal thickness as a marker of cardiovascular risk.

## 1. Introduction

The complications of hypertension depend not only on the mean blood pressure (BP) but also on its variability (BPV).

Although further studies are still needed to fully understand the actual prognostic role of blood pressure variability (BPV), the correlation between increased BPV and the risk of cardiovascular complications in hypertensive patients is well established. In fact, the European Society of Hypertension (ESH) and the European Society of Cardiology (ESC) have recognized the clinical utility of BPV [1]. Recent studies, on the other hand, suggest that the choroid may serve as an indicator of systemic vascular damage. Today, we have access to new diagnostic tools capable of studying in detail the retinal capillary circulation and, most importantly, the underlying choroidal layer, which constitutes the most important vascular layer of the eye. The choroid is not only the most vascularized tissue in the body, but also the sole tissue responsible for the trophism of the avascular foveal region [2,3].

These studies have been made possible by the increased availability of optical coherence tomography (OCT). The ocular microcirculation is uniquely distinctive in that it can be visualized, represented, and quantified in a non-invasive and direct manner. The non-invasive nature and the increasingly widespread availability of OCT in outpatient clinical settings offer significant potential for the assessment of ocular microcirculation as a window into the health of the systemic cardiovascular system. The aim of our study was to analyze the relationship between short-term BP variability (STBPV) and choroid–retinal thickness in hypertensive patients.

## 2. Materials and Methods

### 2.1. Subjects

A total of 98 Caucasian hypertensive patients, aged between 25 and 75 years, were selected for the study from those attending our Nephrology and Hypertension outpatient clinic according to the exclusion criteria listed below:

- Secondary hypertension (renovascular, malignant, or endocrine hypertension, or hypertension associated with obstructive sleep apnea syndrome);
- Diabetic patients;
- Patients with congenital or hereditary kidney diseases, or severe renal insufficiency (eGFR < 30 mL/min/1.73 m^2^);
- History of ocular diseases (e.g., cataracts) and/or ophthalmic surgery;
- Past or recent history of cerebrovascular events;
- History or clinical signs of heart failure (NYHA II-IV) or coronary artery disease;
- Major non-cardiovascular chronic diseases (COPD, liver cirrhosis);
- Neoplasms.

Each patient provided written informed consent.

#### 2.1.1. Clinical and Laboratory Examination

Each participant underwent a comprehensive medical history review and a thorough physical examination. Subjects who reported quitting smoking for just over a year were considered current smokers. Office blood pressure (OBP) was measured according to the 2023 ESH/ESC recommendations using a validated automatic oscillometric sphygmomanometer (WatchBP Office, Microlife AG, Widnau, Switzerland) [4]. Measurements were taken on both arms, with the average of three consecutive readings obtained after 5 min of rest, in a quiet environment, and in a seated position. The value from the arm with the highest recorded blood pressure was used for a statistical analysis. Hypertension was defined as blood pressure values of 140/90 mmHg or higher, or in patients on antihypertensive medication. Each patient underwent 24 h ambulatory blood pressure monitoring (ABPM) using a validated device (BPLab EC-3H/ABPM system, Russia) [5], with hypertension defined according to the 2023 ESC/ESH guidelines [1].

Anthropometric parameters (weight, height, body mass index [BMI]) and routine blood tests were collected for each patient; LDL cholesterol was calculated using the Friedewald formula.

The estimated glomerular filtration rate (eGFR) was calculated using the CKD-EPI (Chronic Kidney Disease Epidemiology Collaboration) equation [6].

The study was conducted according to the guidelines of the Declaration of Helsinki.

Ethical review and approval were waived due to the type of the study, which is an anonymous observational survey on a dataset. Patients gave consent through the compilation of medical records.

#### 2.1.2. Ophthalmic Examination

Each subject underwent a comprehensive ophthalmic examination, including assessment of intraocular pressure and fundus evaluation. The presence of hypertensive retinopathy was assessed for each patient according to the Keith–Wagener–Baker scale by a physician unaware of the patient’s medical history.

The morphological evaluation of the choroidal–retinal region was performed using Swept-Source Optical Coherence Tomography (SS-OCT), always in the morning during the same temporal interval (10–12 a.m.).

All of the scans were performed by a single operator. Each scan was conducted by a single operator following standardized protocols and subsequently evaluated by two ophthalmology specialists. For each patient, data from a single randomly selected eye were used, as there were no significant morphological differences between the right and left eyes.

**Figure 1:**
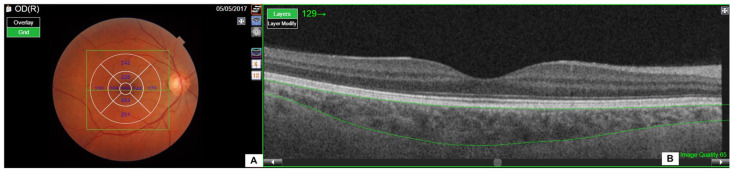
Chorioretinal imaging with the face (**A**) and cross-sectional (**B**) optical coherence tomography.

Choroidal thickness (ChT), defined as the thickness of the layer between the outer surface of the retinal pigment epithelium and the choroid–sclera interface, and retinal thickness (from the inner limiting membrane to the innermost layer of the retinal pigment epithelium) were automatically calculated using specialized SS-OCT software (DRI Triton version 1.04E—1.36.2, Topcon Inc., Tokyo, Japan). The macula was divided into three concentric rings with a diameter of 6 mm centered on the fovea [7]. The inner and outer rings were further divided into four fields, superior, inferior, nasal, and temporal, as shown in Figure 1A from the study by Vadalà et al. [8], which utilized the same evaluation method.

The thicknesses of all nine fields of the choroid were measured in each eye, as well as the average thickness of the four fields of the inner ring (ChT-air: ChT average inner ring) and the outer ring (ChT-aor: ChT average outer ring) separately. The average thickness of all nine choroidal fields (ChT-or: ChT overall ring) and the perpendicular thickness from the retinal pigment epithelium to the choroid–sclera interface (Caliper CSI) were also calculated (Figure 1B).

### 2.2. Statistical Analysis

The statistical analysis was conducted using the IBM-SPSS package (version 26).

Continuous variables are expressed as the mean ± standard deviation or, in the case of non-Gaussian distribution, as medians with interquartile ranges. Differences between groups were analyzed using Student’s *t*-test for unpaired data. Categorical variables were compared using the Chi-square test or Fisher’s exact test, where appropriate. The relationship between variables was assessed using Pearson’s correlation coefficient and a simple and stepwise multiple linear regression analysis. In the multivariate models, the dependent variables included the overall mean of the 9 choroidal fields, the mean of the inner and outer choroidal rings, the overall mean of the 9 retinal fields, and the mean of the outer and inner retinal rings. Independent variables were those found to be associated with the described variables in univariate analyses. A *p*-value of <0.05 was considered statistically significant.

## 3. Results

In this retrospective study, 98 subjects were included, of whom 71% were male. Table 1 displays the demographic and clinical characteristics of the entire study population, as well as those in the two distinct groups based on sex.

Table 2 presents the values and variability indices for both clinical blood pressures and those measured through ABPM. Nighttime mean diastolic blood pressure, the daytime ARV index of blood pressure, and the nighttime heart rate dip were found to be higher in the male population compared to the female population, while nighttime mean heart rate, in contrast, was higher in women.

No significant differences were observed in chorioretinal thickness between men and women (see Table 3). Similarly, subjects receiving treatment did not show significant differences in any of the chorioretinal thickness measurements compared to those not receiving treatment. Additionally, no differences were found between groups based on smoking habits.

Table 4 highlights the univariate correlations between choroidal and retinal thicknesses and various anthropometric and clinical parameters. Among these, the most significant correlations were observed with age, LDL cholesterol, eGFR, and albuminuria.

Table 5 and Table 6 present the bivariate correlations between choroidal and retinal thicknesses and both clinical and 24 h ambulatory blood pressure values, as well as between the former and blood pressure variability indices. Notably, the correlations with the daytime, nighttime, and 24 h ARV indices stand out due to their high statistical significance.

**Figure 2:**
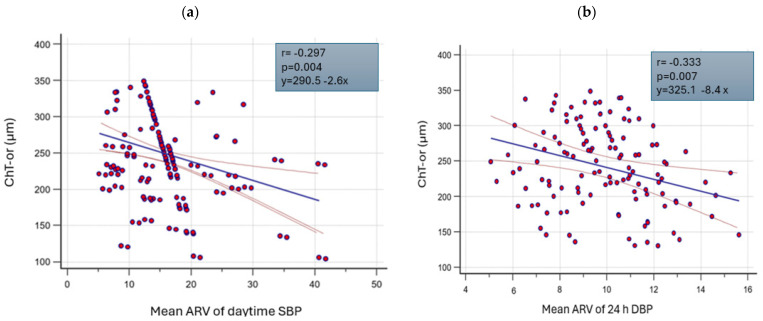
(**a**) Relationship between daytime diastolic blood pressure variability and overall choroidal thickness. (**b**) Relationship between 24 h diastolic blood pressure variability and overall choroidal thickness.

Figure 2a illustrates the relationship between the daytime systolic pressure ARV and choroidal thickness (ChT-or), while Figure 2b depicts the relationship between ChT-or and the 24 h diastolic pressure ARV.

These relationships remain significant even after correction for multiple confounding factors in a multiple linear regression analysis aimed at identifying variables independently associated with choroidal thickness (see Table 7). Additional independent associations of choroidal thickness with other parameters are also shown in Table 7.

In multivariate analyses, the association between choroidal thickness and certain variables, including log albumin, the daytime diastolic blood pressure (DBP) mean, and the daytime DBP ARV mean, remained statistically significant even after accounting for confounding factors such as age, total cholesterol, hemoglobin, and estimated glomerular filtration rate (eGFR).

All variables associated with retinal thickness in univariate analyses lost statistical significance in multivariate analyses.

## 4. Discussion

The notion that the retinal vasculature can serve as a window for observing the body’s microcirculation in vivo—easily, safely, and repeatedly—has been recognized for over a century and is part of the foundational knowledge of every physician. However, the relationships between extraocular vascular diseases and alterations in the choroid remain less explored, despite the choroid being a highly vascularized structure that plays a crucial role in regulating the metabolism and volume of the eye. For a long time, the structural assessment of the choroid in vivo was constrained by its deep location, behind the retinal pigment epithelium (RPE), making it challenging to obtain reliable images. In recent years, however, our understanding of the choroid has been significantly advanced with the introduction of optical coherence tomography (OCT).

We identified a statistically significant relationship between choroidal thickness and blood pressure variability, which remained robust even after a multivariate analysis. No differences in choroidal thickness were observed based on gender, smoking habits, or the use of antihypertensive therapy. In addition to age, other clinical and biochemical factors were found to be strongly associated with choroidal alterations, with albuminuria showing the most significant association.

Several studies have highlighted a correlation between choroidal thickness and albuminuria, suggesting that an increase in choroidal thickness may be associated with high levels of albuminuria [9,10,11]. These results can be interpreted in various ways.

First, the increased choroidal thickness could reflect vascular damage occurring not only at the retinal level, but also at the renal level. In fact, both districts could suffer similar damage due to systemic factors, such as hypertension and inflammation.

Furthermore, the observed correlation could suggest systemic inflammatory activity, since conditions leading to increased albuminuria are often associated with inflammatory processes capable of also influencing choroidal tissue [12].

Finally, it is important to consider the possible impairment of blood perfusion, which can occur at both the renal and retinal levels. Alterations in choroidal perfusion can therefore lead to changes in choroidal thickness, manifesting as increased thickness in the presence of albuminuria.

In multivariate analyses, the association between choroidal thicknesses and some variables including albuminuria, mean daytime DBP, and mean ARV of daytime DBP remained statistically significant even for confounding factors.

While the univariate analysis revealed associations between certain blood pressure variables and retinal thickness changes, these lost statistical significance in the multivariate analysis after adjusting for confounding factors.

Previous studies have yielded inconsistent results regarding the relationship between blood pressure and chorioretinal thickness. Balmforth et al. [12] found no significant association between systolic or diastolic blood pressure and choroidal or retinal thicknesses, with no differences observed when comparing hypertensive patients to normotensive controls. In contrast, Akay et al. [13] and Masis et al. [14] reported that the choroid was thinner in hypertensive patients compared to non-hypertensive individuals. Notably, renal function was not assessed in either of these studies, and thus its potential influence on the relationship between choroidal thickness and blood pressure was not considered.

Gök et al. [15], recently, in 116 hypertensive patients who underwent 24 h ABPM and SD-OCT, did not find any significant relationship of choroidal thickness or the average of BP readings recorded over 24 h with circadian BP variability.

Our findings can be explained in several ways. The thinning of the choroid observed in individuals with higher blood pressure variability might be the result of mechanical damage to this vascular structure, potentially caused by increased stiffness of large arteries. Previous studies have confirmed that such arterial stiffness is associated with heightened blood pressure variability indices [16,17,18]. It is well known that increased stiffness in large arteries amplifies the transmission of pulsatile energy to the microcirculation, particularly in low-impedance areas such as the kidney, brain, and choroid—the latter being the most vascularized among them.

Chronic exposure of microcirculatory vessels to elevated pulsatile energy leads to vasoconstriction in resistance vessels and, consequently, structural damage.

The observed association between the ambulatory arterial stiffness index (ARV) and choroidal thickness, in contrast to the lack of correlation between other variability indices and choroidal thickness, confirms the greater prognostic significance of ARV, as highlighted in previous studies. This is consistent with the considerations discussed in the introduction. However, the correlations between the estimated glomerular filtration rate (eGFR) and chorioretinal thicknesses, demonstrated in earlier studies [8,12,19], were not confirmed in our multivariate analysis. This discrepancy may be due to the overall better renal function in our study population compared to those in previous studies [19] (mean eGFR = 69.99 mL/min/1.73 m^2^).

The loss of statistical significance in the multivariate analysis regarding associations between the heart rate, day/night systolic blood pressure (SBP) ratio, and retinal thicknesses suggests that the retinal area might be less sensitive to early damage resulting from increased blood pressure compared to the choroid. Therefore, changes in choroidal thickness could potentially serve as a more sensitive marker of organ damage than changes in retinal thickness.

Choroidal thinning may also reflect a more generalized microcirculatory rarefaction, leading to increased peripheral resistance. This could exacerbate the magnitude of arterial wave reflection phenomena, thereby increasing arterial stiffness and pulsatility, and ultimately blood pressure variability.

An additional noteworthy finding is the positive association between total cholesterol or LDL cholesterol and certain choroidal thicknesses. Although seemingly paradoxical, this result aligns with the findings of Kim et al. [20], who demonstrated that lipid deposition and subsequent vascular hypertrophy in an experimental model led to increased choroidal thickness.

It is important to emphasize that the cross-sectional design of our study does not allow for the establishment of causal links between increased blood pressure variability and choroidal thinning. It is also possible that both choroidal thinning and increased blood pressure variability are manifestations of vascular damage caused by pathological factors simultaneously affecting the choroid and blood vessels. Consequently, further prospective studies with larger sample sizes are needed to determine the causal direction between the variables we have associated.

SS-OCT measurements can offer a promising tool to integrate advanced ocular imaging into the routine management of hypertension, compared to the Keith–Wagner classification, for example, which is less sensitive to early microcirculatory changes characteristic of the initial stages of hypertensive disease and is also affected by inter-observer variability. Unlike these advanced methods, the Keith–Wagner classification has more limited prognostic value and, by focusing solely on the retina, does not provide data on the choroid, which, as we have seen, appears to offer important insights into systemic vascular damage.

By providing high-resolution, in vivo assessments of retinal and choroidal structure, SS-OCT can aid in the early detection of hypertensive changes in the ocular microvasculature, such as narrowing of the retinal arteries, microaneurysms, or choroidal thickening. These findings could serve as valuable biomarkers for monitoring hypertension-related organ damage and the risk of cardiovascular events. Regular screening with SS-OCT could complement traditional blood pressure measurement, allowing for a more comprehensive approach in the management of hypertensive patients, particularly in assessing the impact of hypertension on the retinal and choroid vasculature. Furthermore, the non-invasive nature of SS-OCT and its ability to detect subtle changes over time make it a useful tool for monitoring the progression of hypertension and evaluating the effectiveness of therapeutic interventions.

If these causal relationships are confirmed by future prospective studies, and if it is demonstrated that choroidal thickness could serve as a more advantageous biomarker of systemic vascular damage compared to currently used cardiovascular markers, it would be reasonable to propose the use of SS-OCT (Swept-Source Optical Coherence Tomography) in clinical practice for determining choroidal thickness.

This could enhance the predictive power of cardiovascular risk stratification and, consequently, improve patient prognoses.

## 5. Conclusions

Our study demonstrated a statistically significant correlation between the reduction in choroidal thickness and the increase in certain blood pressure variability indices.

The loss of statistical significance in the multivariate analysis of variables associated with retinal thickness, which were significant in the bivariate analysis, suggests that the retinal region is less sensitive than the choroid to early damage secondary to elevated blood pressure. Therefore, we could consider changes in choroidal thickness as a more sensitive and early marker of organ damage compared to changes in retinal thickness.

Prospective studies are necessary to better assess the actual prognostic role of choroidal thickness alterations in hypertensive patients. This would allow for the proposal of SS-OCT use in clinical practice to improve cardiovascular risk prediction and the prognosis of hypertensive patients.

## Figures and Tables

**Table 1:** Demographic and clinical characteristics of the whole sample and the two subgroups distinguished by sex.

	Whole Population (*n* = 70)	Men (*n* = 70)	Women (*n* = 28)	*p*
Age (Years)	49 ± 12	53 ± 12	47 ± 12	ns
Antihypertensive Drugs (%)	69	42.6	57	ns
Smokers (%)	32	29	71	ns
BMI (kg/m^2^)	28 ± 5	29 ± 5	27 ± 5	ns
Total Cholesterol (mg/dL)	188 ± 33	188 ± 36	188 ± 28	ns
HDL Cholesterol (mg/dL)	47 ± 13	53 ± 13	45 ± 12	ns
LDL Cholesterol (mg/dL)	117 ± 29	114 ± 29	115 ± 30	ns
Triglycerides (mg/dL)	107 (76–164)	110 (72–158)	101 (78–143)	ns
Blood Glucose (mg/dL)	98 ± 18	101 ± 19	93 ± 14	ns
Hemoglobin (g/dL)	14.4 ± 1	15.1 ± 1	13 ± 1	<0.01
Uricemia (mg/dL)	7 ± 5	6 ± 1.4	8.7 ± 8.3	ns
Serum Creatinine (mg/dL)	1.01 ± 0.4	1.04 ± 0.3	0.95 ± 0.7	ns
Microalbuminuria (mg/24 h)	30 (4.8–77.5)	30 (4–42)	30 (12–237)	ns

ns = not significant.

**Table 2:** Clinical and ambulatory 24 h mean blood pressure values and indices of BPV.

	Whole Population (*n* = 70)	Men (*n* = 70)	Women (*n* = 28)	*p*
**OBP**				
cSBP (mmHg)	138 ± 14	138 ± 14	139 ± 14	ns
cDBP (mmHg)	86 ± 11	87 ± 12	85 ± 9	ns
cPP (mmHg)	52 ± 10	51 ± 10	54 ± 11	ns
cMAP (mmHg)	104 ± 11	104 ± 12	103 ± 10	ns
**ABPM**				
Mean 24 h SBP (mmHg)	130 ± 14	130 ± 14	130 ± 14	ns
Mean Day SBP (mmHg)	134 ± 15	134 ± 15	134 ± 14	ns
Mean Night SBP (mmHg)	119 ± 15	120 ± 15	118 ± 14	ns
Day/Night SBP (mmHg)	1.12 ± 0.09	1.12 ± 0.09	1.13 ± 0.09	ns
Mean 24 h DBP (mmHg)	84 ± 9	84 ± 8	82 ± 9	ns
Mean Day DBP (mmHg)	87 ± 9	88 ± 9	86 ± 10	ns
Mean Night DBP (mmHg)	76 ± 9	77 ± 9	73 ± 9	0.04
Day/Night DBP (mmHg)	1.16 ± 0.11	1.14 ± 0.11	1.19 ± 0.12	ns
Mean 24 h PP (mmHg)	46 ± 12	45 ± 12	48 ± 11	ns
Mean Day PP (mmHg)	47 ± 12	46 ± 12	48 ± 12	ns
Mean Night PP (mmHg)	43 ± 11	42 ± 11	45 ± 11	ns
Day/Night PP (mmHg)	0.45 ± 0.55	0.50 ± 0.54	0.31 ± 0.55	ns
**BP variability indices**				
Mean 24 h ARV SBP (mmHg)	12 ± 2.6	13 ± 2.4	12 ± 3.2	ns
Mean 24 h ARV DPB (mmHg)	10 ± 3.4	10 ± 2.4	9 ± 2.5	ns
Mean Day ARV SBP (mmHg)	15 ± 3.6	15 ± 3.3	14 ± 4	0.05
Mean Night ARV SBP (mmHg)	16 ± 7	15 ± 6.4	16 ± 8.6	ns
Mean Day ARV DBP (mmHg)	10 ± 2.3	10 ± 2.4	10 ± 2	ns
Mean Night ARV DBP (mmHg)	13 ± 4.6	13 ± 4	13 ± 6	ns
SD Day SBP (mmHg)	14 ± 4.5	14 ± 3.7	15 ± 6.2	ns
SD Night SBP (mmHg)	12 ± 3.5	12 ± 3.5	12 ± 3.5	ns
SD Day DBP (mmHg)	11 ± 3,6	11 ± 3,7	11 ± 3.4	ns
SD Night DBP (mmHg)	10 ± 3	10 ± 3.1	10 ± 2.7	ns
SD Day PP (mmHg)	13 ± 5.6	13 ± 6.1	13 ± 4	ns
SD Night PP (mmHg)	10 ± 5.2	10 ± 5.5	11 ± 4.4	ns
SD Day HR (bpm)	9 ± 3.2	9 ± 2.8	9 ± 4	ns
SD Night HR (bpm)	7 ± 3	7 ± 3.1	7 ± 2.72	ns
24 h Weighted Bilo SBP	14 ± 4.9	14 ± 3	14 ± 7.2	ns
24 h Weighted Bilo DBP	12 ± 4	12 ± 3	12 ± 5.7	ns

Abbreviations—SBP: systolic blood pressure; DBP: diastolic blood pressure; PP: pulse pressure; HR: heart rate. ns = not significant.

**Table 3:** Choroidal and Retinal Thicknesses Measured by SS-OCT in the Overall Population and in the two subgroups.

	Whole Population (*n* = 70)	Men (*n* = 70)	Women (*n* = 28)	*p*
**Choroidal thicknesses**				
ChT-c (µm)	259.5 ± 73.2	265.3 ± 73.3	245.5 ± 72.7	ns
ChT-air (µm)	235.1 ± 58.2	239.5 ± 60.5	224.6 ± 51.6	ns
ChT-aor (µm)	253.6 ± 65.5	258.7 ± 68.2	242.7 ± 58	ns
ChT-or (µm)	249.7 ± 61.4	243.1 ± 63.6	241.7 ± 55.8	ns
Caliper CSI (µm)	272.2 ± 72.9	274.9 ± 77.1	267.5 ± 67	ns
**Retinal thicknesses**				
RetT-air (µm)	279 ± 92.6	275.6 ± 85.1	287.2 ± 110.1	ns
RetT-aor (µm)	308 ± 19.5	309.7 ± 21.5	303.8 ± 12.5	ns
RetT-or (µm)	290.6 ± 46.4	289.7 ± 41.8	292.7 ± 56.9	ns

Abbreviations—ChT-c: ChT average central ring; ChT-air: ChT average inner ring; ChT-aor: ChT average outer ring; ChT-or: ChT overall ring; Caliper CSI: choroid–sclera interface; RetT-air: Ret average inner ring; RetT-aor: Ret average outer ring; RetT-or: Ret overall ring. ns = not significant.

**Table 4:** Bivariate relationships between choroidal and retinal thicknesses and clinical and laboratory variables.

	Age	Total Cholesterol	LDL Cholesterol	Glycemia	Hemoglobin	Serum Creatinine	eGFR	Log Albuminuria
	r	r	r	r	r	r	r	r
**Choroidal thicknesses**								
ChT-c (µm)	−0.364 **	0.263 **	0.287 **	ns	ns	ns	0.212 *	−0.361 ^
ChT-air (µm)	−0.455 **	ns	0.228 *	−0.288 *	ns	−0.241 *	0.346 **	−0.303 *
ChT-aor (µm)	−0.445 **	0.248 *	−0.353 *	−0.289 *	0.238 *	Ns	0.264 *	−0.389 ^^
ChT-or (µm)	−0.465 **	0.248 *	0.039*	−0.290 *	ns	−0.261 *	0.358 **	−0.370 ^
**Retinal thicknesses**								
RetT-air (µm)	ns	0.310 *	0.278 ^	ns	ns	ns	ns	ns
RetT-aor (µm)	ns	ns	ns	ns	ns	ns	ns	ns
RetT-or (µm)	ns	ns	0.269 **	ns	ns	ns	ns	ns

Abbreviations—ChT-c: ChT average central ring; ChT-air: ChT average inner ring; ChT-aor: ChT average outer ring; ChT-or: ChT overall ring; RetT-air: Ret average inner ring; RetT-aor: Ret average outer ring; RetT-or: Ret overall ring; eGFR: estimated glomerular filtration rate. * *p* ≤ 0.05; ** *p* ≤ 0.01; ^ *p* ≤ 0.005; ^^ *p* ≤ 0.001; ns = not significant.

**Table 5:** Bivariate Relationships Between Choroidal and Retinal Thicknesses and clinical and laboratory variables.

	cPP	cMAP	c-HR	Mean Night SBP	Mean Daytime DBP	Day/Night SBP	SD Night SBP	SD Day PP	SD Day HR
	r	r	r	r	r	r	r	r	r
**Choroidal thicknesses**									
ChT-c (µm)	ns	0.203 *	ns	ns	ns	ns	ns	ns	ns
ChT-air (µm)	ns	ns	ns	ns	ns	ns	ns	ns	ns
ChT-aor (µm)	−0.231 *	ns	ns	ns	0.272 **	ns	ns	0.107 *	−0.232 **
ChT-or (µm)	ns	ns	0.253 **	ns	0.217 *	ns	ns	ns	ns
Caliper CSI (µm)	−0.227 *	ns	ns	−0.230 *	ns	ns	ns	ns	ns
**Retinal thicknesses**									
RetT-air (µm)	ns	ns	ns	ns	ns	ns	ns	ns	ns
RetT-aor (µm)	ns	ns	ns	ns	ns	−0.226 *	ns	ns	ns
RetT-or (µm)	ns	ns	0.204 *	ns	ns	ns	ns	ns	ns

Abbreviations—ChT-c: ChT average central ring; ChT-air: ChT average inner ring; ChT-aor: ChT average outer ring; ChT-or: ChT overall ring; RetT-air: Ret average inner ring; RetT-aor: Ret average outer ring; RetT-or: Ret overall ring. * *p* ≤ 0.05; ** *p* ≤ 0.01; ns = not significant.

**Table 6:** Bivariate Relationships Between Choroidal and Retinal Thicknesses, 24 h Clinical and Ambulatory Blood Pressure Values, and Blood Pressure Variability Indices.

	Mean 24 h DBP	Mean 24 h ARV SBP	Mean 24 h ARV DBP	Mean Daytime ARV SBP	Mean Night ARV SBP	Mean Daytime ARV DBP
	r	r	r	r	r	r
**Choroidal thicknesses**						
ChT-c (µm)	ns	ns	−0.331 **	ns	−0.311 **	−0.268 **
ChT-air (µm)	0.222 *	−0.252 *	−0.316 **	ns	−0.290 ^	−0.266 **
ChT-aor (µm)	ns	ns	−0.320 **	ns	−0.283 **	−0.272 **
ChT-or (µm)	ns	ns	−0.333 **	0.208 *	−0.295 ^	−0.297 ^
Caliper CSI (µm)	ns	ns	−0.365 **	ns	ns	−0.278 *
**Retinal thicknesses**						
RetT-air (µm)	ns	ns	ns	ns	ns	ns
RetT-aor (µm)	ns	ns	ns	ns	ns	ns
RetT-or (µm)	ns	ns	ns	ns	ns	ns

Abbreviations—ChT-c: ChT average central ring; ChT-air: ChT average inner ring; ChT-aor: ChT average outer ring; ChT-or: ChT overall ring; RetT-air: Ret average inner ring; RetT-aor: Ret average outer ring; RetT-or: Ret overall ring; eGFR: estimated glomerular filtration rate. SBP: systolic blood pressure; DBP: diastolic blood pressure; PP: pulse pressure. * *p* ≤ 0.05; ** *p* ≤ 0.01; ^ *p* ≤ 0.005. ns = not significant.

**Table 7:** Variables independently associated with choroidal thicknesses.

Dependent Variable	ChT-or				ChT-c				ChT-aor				ChT-air			
Regressors																
	B	S.E.	β	*p*	B	S.E.	β	*p*	B	S.E.	β	*p*	B	S.E.	β	*p*
Log-albuminuria	−39	9.2	−0.53	<0.001	−46.6	10.1	−0.56	<0.001	−37.5	8.9	−0.52	<0.01	−37	10	−0.47	<0.001
Mean daytime DBP	2.14	0.7	0.32	0.008	2.42	0.8	0.32	0.007	1.92	0.7	0.29	0.016	2.9	0.8	0.41	<0.001
Mean ARV day DBP	−10.9	3.13	−0.39	0.001	−10.7	3.5	−0.34	0.004	−9.3	3.1	−0.35	0.005	−10.2	3.2	−0.36	0.003

Abbreviations—ChT-c: ChT average central ring; ChT-air: ChT average inner ring; ChT-aor: ChT average outer ring; ChT-or: ChT overall ring; B: unstandardized regression coefficient; S.E.: standard error; β: standardized regression coefficient.

## Data Availability

The datasets generated during and/or analyzed during the current study are available from the corresponding author on reasonable request.
